# A 3D scaling law for supravalvular aortic stenosis suited for stethoscopic auscultations

**DOI:** 10.1016/j.heliyon.2024.e26190

**Published:** 2024-02-15

**Authors:** Ahmed M. Ali, Aly A. Ghobashy, Abdelrahman A. Sultan, Khalil I. Elkhodary, Mohamed El-Morsi

**Affiliations:** Department of Mechanical Engineering, The American University in Cairo, 11835 New Cairo, Egypt

**Keywords:** Aorta, SVAS, Stenosis, Hemoacoustics, Phonocardiography

## Abstract

In this study a frequency scaling law for 3D anatomically representative supravalvular aortic stenosis (SVAS) cases is proposed. The law is uncovered for stethoscopy's preferred auscultation range (70-120 Hz). LES simulations are performed on the CFD solver Fluent, leveraging Simulia's Living Heart Human Model (LHHM), modified to feature hourglass stenoses that range between 30 to 80 percent (mild to severe) in addition to the descending aorta. For physiological hemodynamic boundary conditions the Windkessel model is implemented via a UDF subroutine. The flow-generated acoustic signal is then extracted using the FW-H model and analyzed using FFT. A preferred receiver location that matches clinical practice is confirmed (right intercostal space) and a correlation between the degree of stenosis and a corresponding acoustic frequency is obtained. Five clinical auscultation signals are tested against the scaling law, with the findings interpreted in relation to the NHS classification of stenosis and to the assessments of experienced cardiologists. The scaling law is thus shown to succeed as a potential quantitative decision-support tool for clinicians, enabling them to reliably interpret stethoscopic auscultations for all degrees of stenosis, which is especially useful for moderate degrees of SVAS. Computational investigation of more complex stenotic cases would enhance the clinical relevance of this proposed scaling law, and will be explored in future research.

## 1 Introduction

**Figure 1:**
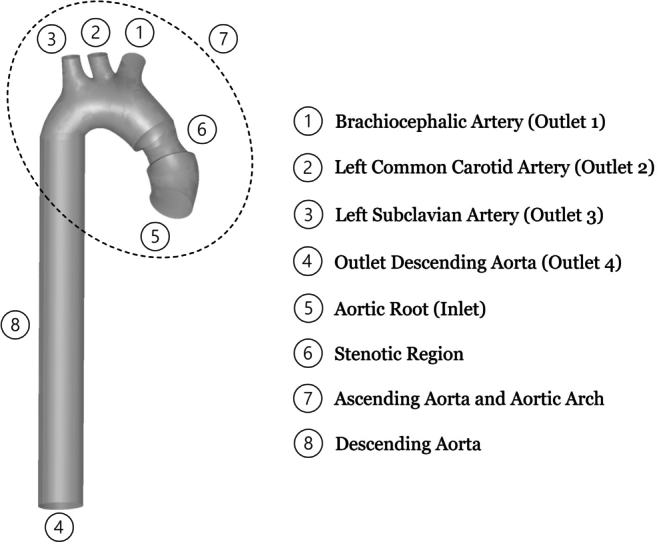
Domain of Interest, simplified anatomy of human Aorta.

Supravalvular aortic stenosis (SVAS) is a congenital heart disease which involves the narrowing of the aorta above the aortic valve at the sinotubular junction [1]. The disease prevails in 8% to 14% of aortic stenosis cases, and is classified as one of three: hypoplasia of the aortic arch, hourglass, or membranous. Hourglass SVAS is the most prevalent [2], and will be the focus of this study. It is considered as a case of left ventricular outflow tract obstruction [3] associated with cardiac diseases, such as Williams syndrome [4], infantile hypercalcemia, or deranged vitamin D metabolism [5], and may affect the entire aortic root apparatus [6]. Hourglass SVAS involves a thickening of the sinotubular junction, in addition to a size increase in the Valsalva sinus and aortic root, which generate its hourglass appearance, see Fig. 1, and obstruct the aortic flow.

Treatment of this defect may include surgery, which is recommended when the pressure gradient is above 30 mmHg. For instance, the Ross procedure [7], which involves replacing the diseased aortic valve with the patient's own pulmonary valve, or other procedures such as resection with end-to-end anastomosis and an application of Y patches [8]. As such, evaluation of this disease is typically performed using hospital-centric techniques, such as echocardiograms [9], electrocardiograms [10], CT [11], MRI [12], and catheterization [13]. Too often, expensive nephrotoxic contrast-enhancing agents are used, which are either invasive or radiative [14]. Another clinical assessment technique that is safer and non-invasive is phonoangiography, which requires a computer-assisted device fitted with advanced sensors to identify *bruits*, i.e., changes in the sound of blood as it flows through a narrowed portion of an artery [15]. However, such investigations do not typically quantify the exact degree of stenosis but can classify it as mild, moderate, or severe (Azimpour et al. [16], Sun et al. [17], Duncan et al. [18], Bakhshaee et al. [19], Watrous et al. [20], Semmlow et al. [21]).

An under-utilized clinical assessment technique (for stensois) is cardiac auscultation. Auscultation involves the acquisition of mechanical vibrations from the surface of the patient's body, which are within the frequency range of audible sound, using a stethoscope [22]. When the sound generated is due to abnormal blood flow, murmurs can be heard and interpreted by an experienced cardiologist. Although this diagnostic tool has been in use for many years [23], its utility has declined over past years due to its dependency on the physicians' experience [24]. Specifically, a low diagnostic accuracy was observed to be of international concern [25]. Generally, the underlying flow phenomena that lead to such murmurs are complex and not yet fully understood [26]. Moreover, it remains difficult with today's technology to perform heart murmur auscultations while simultaneously imaging a patient's hemodynamics, to clinically connect the two. As such, there has been a growing interest in *hemoacoustic* research, to experimentally and computationally explore such connections.

Several studies have sought a deeper understanding of the relationship between murmurs and hemodynamics via experimental setups. For instance, it was observed that murmurs are caused by periodic fluctuations in the downstream (post-stenotic) wake of the flow, which emit most of the acoustic energy, but not due to the turbulent nature of the blood flow [27]. Another study of the relationship between hemodynamic and acoustic processes was conducted on an in-vitro setup created to investigate the variation in the stenosis diameter and length in relation to the strength of the flow-generated acoustic signals [28]. Their findings confirmed that the acoustic power spectrum increases measurably with decreasing stenosis diameter, while increasing the length of the occlusion produces a measurable drop in the sound level, indicating a critical length after which the audible difference between healthy and diseased vessels is lost. Further theoretical-cum-experimental investigations into the vibration of silicone tubes (representing blood vessels) with axisymmetric occlusions were performed, which noted discrepancies between hemodynamic theory and observed flow phenomena that may be ascribed tube compliance and vibration, and for failing to capture the viscoelastic nonlinearity of the vessel [29].

Such empirical studies succeeded in connecting the key acoustic and hemodynamic *phenomena* of stenosis. Nevertheless, connecting their underlying *mechanisms* remains elusive, due to their evolutionary complexity at the phenomenological scale. In-silico modeling was thus proposed as a potential solution by many researchers (e.g., Jiang et al. [30], Oshin et al. [31], Richez et al. [32], Morris et al. [33], and Bailoor et al. [26]). Such works illustrate the use of different computational methods to shed light on a variety of underlying hemodynamic and hemoacoustic mechanisms in the cardiovascular system. Of special interest to this manuscript is the computational method known as *computational fluid dynamics* (CFD), which has shown great promise in simulating flow structures in the aorta with an accuracy matching that of clinical 4D-MRI [34]. CFD has also been applied to the simulation of proposed surgical interventions for virtual surgical planning [35]. The accuracy of CFD simulations is frequently improved by complementing the model with 4D-MRI-extracted boundary conditions [36], implemented in the form of a Windkessel model [37]. Such physiologically inspired boundary conditions capture the relevant behavior of blood flow in distal arteries [38] by means of an electrical circuit analogy [39]. With such a computational setup, helical flow patterns in the aorta [40], and thrombus formation in an aneurysmatic aorta [41], could be successfully captured. Likewise, hemodynamic parameters of the aorta subjected to different aortic valve pathologies could be virtually varied and investigated [42]. Moreover, blood flow quality post surgical repair of coarctation [43] or valve-conserving operations could be assessed in depth by clinicians [44].

CFD alone, however, cannot yield insights into hemoacoustics. It must be supplemented with a computational acoustic analysis of the pressure fields solved for by CFD. Indeed, several studies computationally investigated the relationship between bruits and hemodynamics. For instance, Borisyuk [45] developed an acoustic model for which a receiver placed on the patient's chest was used the obtain noise propagation due to the fluctuation of the pressure field associated with their blood flow. The model was used to compare the predictive performance of theoretical wall-pressure models, viz. Ffowcs Williams [46], Corcos [47], Smol'yakov and Tkachenko [48], for a simplified stenosed artery in two-dimensions. The study thus provided an excellent benchmark for hemoacoustic analyses. Seo et al. [49] further investigated the relationship between arterial bruits and arterial wall pressure for a CFD domain consisting of a channel with a one-sided constriction, capturing the flow-induced sound using linearized perturbed compressible equations (LPCE). Their analysis confirmed that acoustic fluctuations generated by blood flow have a stronger intensity and a higher frequency content when the constriction degree increases. The study however does not feature an anatomical geometry that can account for clinically observed helical and retrograde secondary arterial flow [50].

Seo et al. [51] later developed a framework for virtual echocardiography, phonocardiography, and a multiphysics blood-flow simulation that integrates CFD with hemoacoustics. This work features a patient-specific geometry, segmented from a CT scan, which includes the left ventricle, left atrium, aorta, and the mitral and aortic valves. As such, they were able to determine correlations between cardiographic output parameters and blood flow dynamics for a specific patient. Building on this framework, Seo et al. [52] improved their predictions by applying a sharp-interface immersed boundary method in their multiphyiscs framework along with a three-dimensional elastic wave equation to a simplified model of an artery (to better capture flow-induced bruits). However, their findings were limited by ignoring the pulsatility of blood flow. Khalili et al. [53] took flow time-variation into consideration and demonstrated that bruits strongly relate to the time-derivative of the pressure field acting at the arterial wall, downstream the stenosis. Importantly, their analysis highlighted the necessity of accounting for the descending aorta in the flow domain when analyzing the acoustics of SVAS.

The analysis of simulated bruits was elaborated on in several other computational studies (e.g., Tse et al. [54], Zhu et al. [55], and Bailoor et al. [26]). All these studies rely on the concept of the *break frequency*, i.e., a frequency that marks a sudden change in spectral power trends, consistent with in-vitro and clinical phonoangiography. The studies confirm that the bruits' break frequency typically falls beyond the recommended range of stethoscopic auscultation (70 - 120 Hz), which limits the scope of applicability of the resulting scaling laws. As such, a different computational approach to finding a scaling law is needed, if the objective is to quantify bruits using stethoscopy. In our recent work [56] a replacement to the break frequency concept was introduced, which was shown to rely on the dominant frequency of the von Karman street, and to always lie within the stethoscopic range for all clinical degrees of stenosis (mild to severe). The study was confined to two-dimensional cases of stenosis, however.

In this study, a scaling law is thus proposed for anatomically representative 3D SVAS cases, which is shown to be well suited for stethoscopic auscultation (and clinically consistent). CFD simulations are performed on several hourglass SVAS models building on Simulia's LHHM geometry per Fig. 1. In the Methods section the details of CFD model development are outlined, advancing the state-of-the-art in the literature in terms of pre-processing, simulation setup, and acoustic post-processing. The Results and Discussion section then outlines the simulated hemodynamic flow fields and uncovers a new linear scaling law. The paper is concluded by discussing the scaling law in relation to multiple clinical stethoscopic signals obtained from various online databases, confirming its great potential as a clinical decision-support tool, with special benefit for moderate stenotic conditions.

## 2 Methods

In this section a computational process is outlined for the building of a library of 3D stenotic geometries relevant to clinical practice and a corresponding database of simulated flow-induced pressure fields and acoustic signals. A scaling law connecting the degree of stenosis to a new acoustic frequency is then defined from these databases. Given the newly identified scaling law, the degree of stenosis in a patient could be accurately and noninvasively determined by clinicians by simply comparing a corresponding frequency extracted from the auscultated signal range to the scaling law. We remark that in the previous study [56], which featured 2D idealized geometries, the designated acoustic frequency was restricted to the von Karman Vortex Street (KVS). In 3D the KVS loses its coherence due to vortex stretching [57], which necessitates a modification to the methodology, as herein proposed.

**Figure 2:**
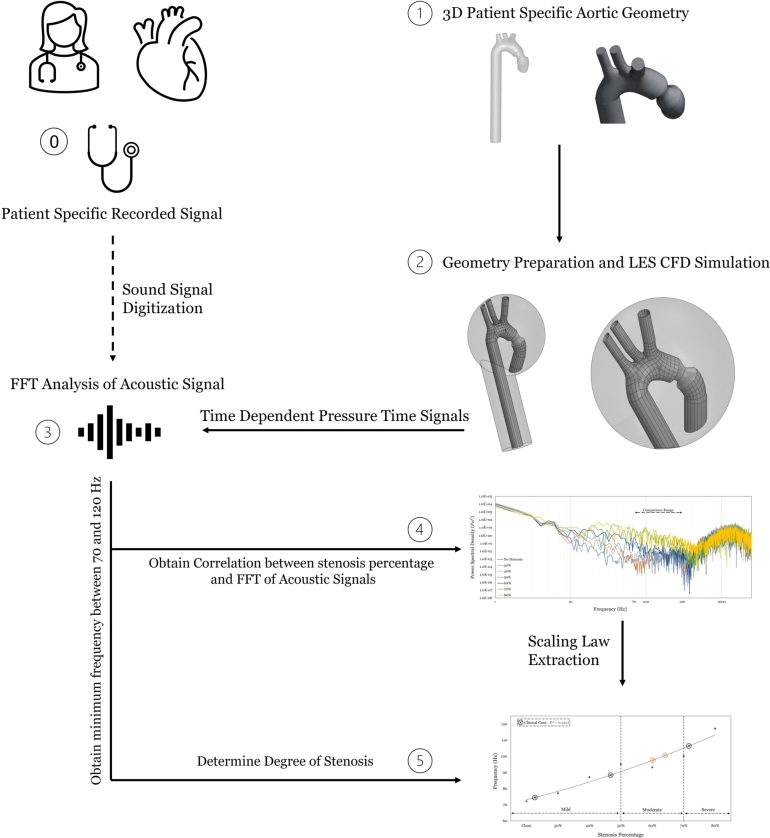
Methodology extending our earlier work [56] to uncover the scaling law between emitted acoustic frequencies in the stethoscopic range (70 - 120 Hz) and the degree of stenosis.

An anatomically accurate 3D geometry of a healthy ascending aorta based on the Living Heart Human Model (LHHM) [58] is adopted. The geometry is herein modified to feature hourglass stenoses of varying degrees; specifically, for percentages varying from 30 percent to 80 percent at increments of 10 percent. Then, CFD simulations are conducted using the Large Eddy Simulation (LES) solver on the commercial software ANSYS Fluent [59]. A velocity wave at the inlet of the ascending aorta is applied to mimic the physiological waveform [60], and a Windkessel model at the outlets is to capture the effect of distal arteries [39]. Using the Ffwocs-Williams Hawkings (FW-H) model, the emitted acoustic signals are obtained at various receiver locations, comparing to clinical practice. Following the acquisition of the optimally located acoustic signal, a Fast Fourier Transform (FFT) is performed in search of a frequency that correlates with stenosis percentage and generates a desired (linear) scaling law. The acoustic frequency range in the analysis is constrained to the one recommended for stethoscopic auscultations, i.e., 70 Hz-120 Hz [61]. This ensures that the scaling law obtained in this 3D study is stethoscopically identifiable, albeit not directly resulting from the KVS (as was in 2D). The scaling law is then verified against clinical auscultations and their reported assessments of stenosis per the NHS classification standards. The proposed method is summarized graphically in Fig. 2, and the following subsections detail it further.

### 2.1 Pre-processing

This subsection details the setup of representative geometric models of 3D stenotic blood vessels (hourglass SVAS), the selection of hemodynamic governing laws and appropriate CFD solvers on ANSYS, and the modeling of physiologically consistent boundary conditions.

#### 2.1.1 Geometric surfaces preparation

**Figure 3:**
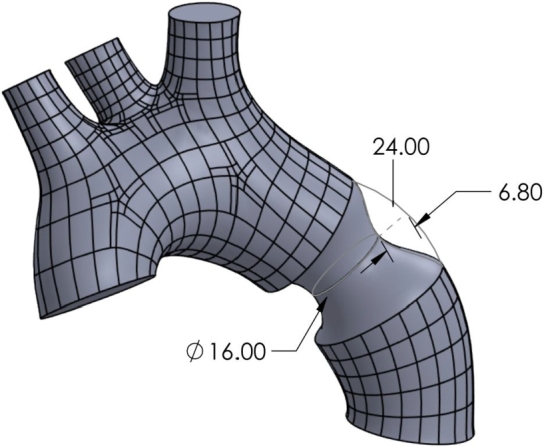
Illustration of an hourglass stenosis for the case of 40%. The stenosed diameter is changed and the other dimensions correspondingly adjusted to capture all cases of stenosis.

The stenosis-free fluid domain simulated in this study is exported from the LHHM (finite element solver Abaqus) into Rhinoceros, a computer-aided design application. This model includes an anatomically accurate representation of the ascending aorta and the aortic arch for a healthy 26 year old male individual. This model is thus modified on Rhinoceros to represent each case of SVAS between 30 percent to 80 percent stenosis, by introducing corresponding hourglass stenoses, as illustrated in Fig. 3.

Then, to attach an anatomically representative descending aorta to the modified LHHM, a vertical vessel is extruded using the cross section of Outlet 4, as indicated in Fig. 1. The importance of modeling the descending aorta is highlighted in [50], if one seeks to successfully capture the development of vortical structures and their emitted acoustics. The length of the descending aorta was determined for this model to be 200 mm, using Equation (1) following [62],

(1) Length=6.76+15.86log⁡(age).

**Figure 4:**
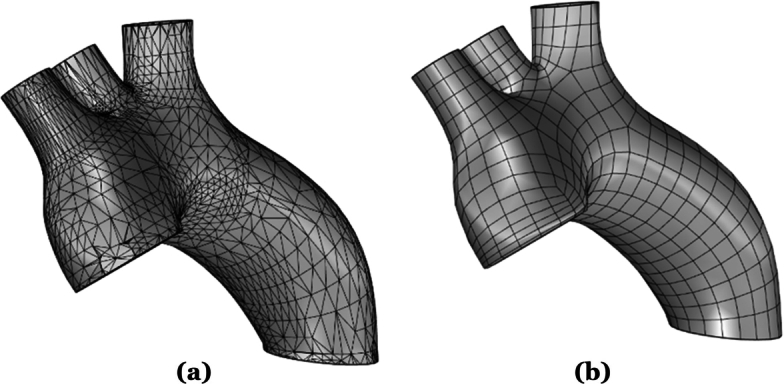
Original aortic arch and ascending aorta per the Living Heart Human Model (LHHM) of Simulia. This model, serves as this study's base geometry, which is modified by adding stenosis and a descending aorta. (a) represents the geometry that was imported directly into Rhinoceros from the LHHM, and (b) depicts the geometry after resurfacing for subsequent hemoacoustic modeling.

Note that the geometry extracted from the LHHM is best exported to Rhinoceros in the Standard Triangle Language (STL) format. However, STL models are composed of small triangles or polygons, which are not directly suited for CFD solvers, causing potential discretization errors. Thus, the STL model depicted in Fig. 4 (a) is converted to Non-Uniform Rational B-Splines (NURBS), by applying Quad resurfacing, which yields Fig. 4 (b). The model is then exported from Rhinoceros to ANSYS Spaceclaim, to patch the resulting geometry prior to CFD modeling, i.e., removing split edges, extra edges, gaps and/or duplicate faces. Then, the volume that encloses the fluid domain is extracted using the volume-extract command in Spaceclaim.

**Figure 5:**
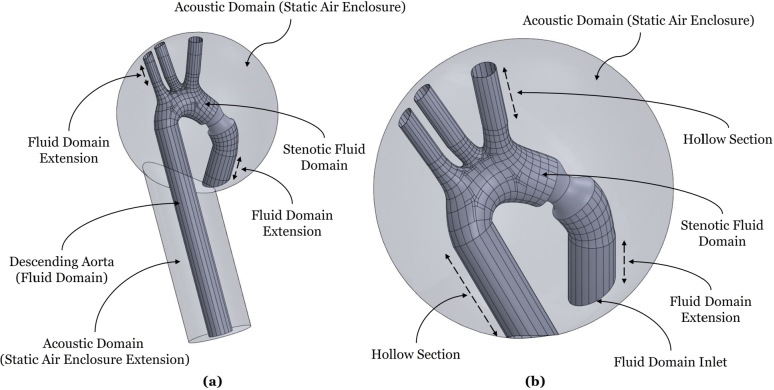
Details of the two models examined in this study. (a) represents the ascending aorta, aortic arch and descending aorta. (b) represents the ascending aorta and aortic arch only. Both models are shown here at 30 percent stenosis. The fluid domains are enclosed within an acoustic domain, which is modeled as static air (ignoring the potential effect of surrounding tissue).

Finally, using the computer-aided design software Solidworks, a volume surrounding the aorta (external to the flow) was added as shown in Fig. 5 (a), (b) to later solve for the flow-generated acoustics. As indicated in the Fig. 5, further extensions were added to the outlets of the ascending aorta to allow for flow development without imposing outlet velocity or pressure profiles, and to avoid boundary effects altering the near flow field.

#### 2.1.2 CFD governing equations

Mass continuity and the incompressible Navier-Stokes equations are used as the governing equations for the CFD simulations, respectively per Equation (2) and Equation (3),

(2) ∇⋅u=0

(3) $$\rho (\partial u/\partial t+(u\cdot \nabla )u)=-\nabla p+\nabla \cdot (\left(\mu +\mu_{t}\right)\left(\nabla u+{\left(\nabla u\right)}^{T}\right)$$

where, *u* is fluid's flow velocity, *ρ* is density, *μ* is kinematic viscosity, and $\mu_{t}$ is the eddy viscosity, or turbulent viscosity, *p* is the pressure, *t* is the time, and *T* is the transpose operator. In this study, the blood density and kinematic viscosity values were assumed to be 1060 ${\text{kg/m}}^{3}$ and 0.00278 kg⋅m/s [56], respectively. The Eddy viscosity is solved for implicitly by ANSYS Fluent [59]. The flow is further considered to be unsteady, and Newtonian. Note that isothermal flow conditions are also applied (constant blood temperature is assumed). Accordingly, the energy equation is not solved for.

The LES model is chosen to capture the turbulent blood flow, since it provides a fair trade-off between computational resources and time [63], while maintaining an excellent capability to resolve the flow's vortical structures [56]. In particular, turbulent flow contains eddies that span a range of sizes and energies. By using an LES model, an explicit resolution of these eddies will depend on mesh size, which results in *explicit filtering*. For the (cascade of) smaller eddies, a sub-grid scale model is used to *implicitly* capture their contribution to the flow field. The Wall-Modeled LES (WMLES S−Ω) sub-grid scale model is herein used since, for channel flow simulations as in this paper, it can be used at the same grid resolution as that adopted for the domain of turbulent flow. It is noted that LES with WMLES S−Ω is best suited for simulations where the wall shear stress is not of considerable interest.

#### 2.1.3 Boundary conditions

**Figure 6:**
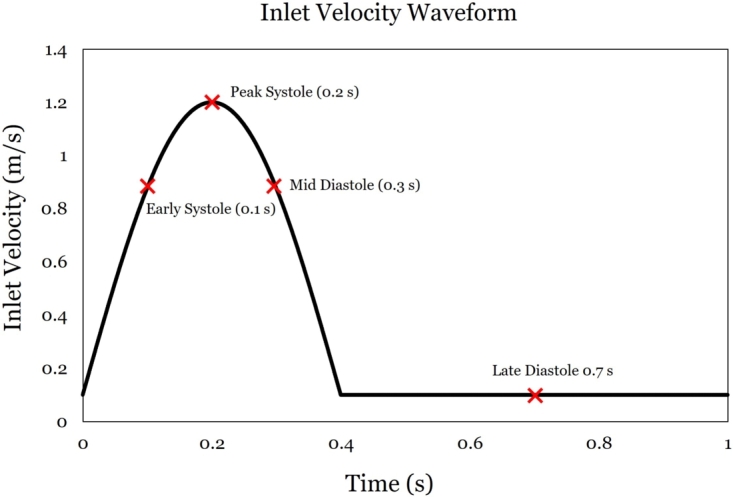
Inlet velocity waveform per [60], implemented through Listing 1. The physiologically relevant instants selected for the in-depth analysis are indicated on the waveform with red crosses.

A no-slip boundary condition was applied along the inner surface of the aorta, assuming it is as a rigid wall [64]. The aortic root was assigned as the inlet boundary condition, where a time-dependent inlet waveform (Fig. 6) was used to simulate pulsating inlet condition, per [60]. This was introduced to the ANSYS solver as an expression shown in Listing 1.

**Listing 1:**
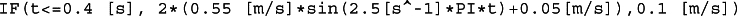
Expression used in Fluent to generate required inlet wave form.

Artificial turbulence (puffs) is added using the ANSYS spectral synthesizer to this inlet waveform, for greater physiological consistency [56]. To define the puffs, the hydraulic diameter is assumed to match the inlet diameter, and the strength of the fluctuations is heuristically set to a value of 5 percent.

Two outlet boundary conditions were compared in this study; a static pressure of 80 mmHg, which corresponds to the diastolic pressure [65], and the physiologically representative Windkessel model [39], [42]. The latter captures the contribution of the arterial tree, beyond the model outlet [66], by including a proximal resistance $R_{{\text{P}}_{i}}$, compliance $C_{i}$, and a distal resistance $R_{{\text{d}}_{i}}$ for each outlet. The overall resistance in the vascular system is known as $R_{t}$. These values were calculated for a representative case according to the following formulae (Equation (4)-(8)) based on the work of [42],

(4) $$R_{\text{total }}=\frac{P}{Q}={\left(\sum_{i}\frac{1}{R_{i}}\right)}^{-1}$$

(5) $$R_{i}=R_{{\text{P}}_{i}}+R_{{\text{d}}_{i}}$$

(6) $$\frac{R_{\text{total }}}{R_{i}}=\frac{A_{i}}{A_{T}}$$

(7) $$\frac{R_{{\text{P}}_{i}}}{R_{{\text{P}}_{i}}+R_{{\text{d}}_{i}}}=0.056$$

(8) $$C_{i}=\frac{C_{t}A_{i}}{A_{t}},$$

where *P* is the mean arterial pressure, *Q* is the mean inlet flow rate, $R_{i}$ is the resistance for each individual outlet which is the summation of proximal $R_{{\text{P}}_{i}}$ and distal $R_{{\text{d}}_{i}}$ resistances for this specific outlet. $A_{i}$ is the cross-sectional area of each individual outlet, $A_{t}$ is the total cross-sectional area of all outlets. By solving Equation (5) and Equation (7), the values of $R_{p}$ and $R_{d}$ are obtained for each outlet. Compliance for each outlet has been calculated similarly using Equation (8), where $C_{t}$ is the total vascular system compliance obtained from [67]. The computed values for $R_{{\text{P}}_{i}}$, $R_{{\text{d}}_{i}}$ and $C_{i}$ for each outlet are presented in Table 1.

**Table 1:** Values for Windkessel's UDF: $R_{{\text{P}}_{i}}$, $R_{{\text{d}}_{i}}$ and *C*_*i*_ are for each outlet.

*A*_*t*_	$6.913e^{\text{-4}}{\text{ m}}^{2}$			
*P*	$1.316e^{\text{-4}}\text{ Pa}$			
*Q*	$1.658e^{\text{-4}}\frac{{\text{m}}^{3}}{\text{s}}$			
*R*_*t*_	$79.373e^{\text{6}}\frac{\text{N s}}{{\text{m}}^{5}}$			
*C*_*t*_	$1.154e^{\text{-8}}\frac{{\text{m}}^{5}}{\text{N}}$			
Variables	Outlet 1	Outlet 2	Outlet 3	Outlet 4

$R_{{\text{P}}_{i}}$	22*e*^6^	33*e*^6^	40*e*^6^	7*e*^6^
$R_{{\text{d}}_{i}}$	378*e*^6^	559*e*^6^	678*e*^6^	134*e*^6^
*C*_*i*_	2.28*e*^-9^	1.54*e*^-9^	1.274*e*^-9^	6.435*e*^-9^

It should be noted that the reverse flow at the outlets is prevented, specifically at systole, with the Windkessel conditions. ANSYS Fluent builds artificial barriers on the faces of the boundary's mesh to keep flow from re-entering the flow domain. Once the flow can no longer re-enter the domain and a favorable pressure gradient is recovered, the artificial walls are removed.

#### 2.1.4 Meshing the fluid domain's interior

**Figure 7:**
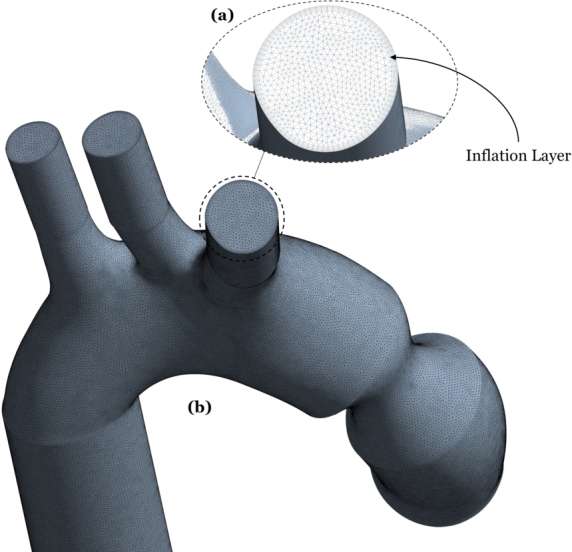
Mesh generated for the 50 percent stenosis case.

**Figure A.1:**
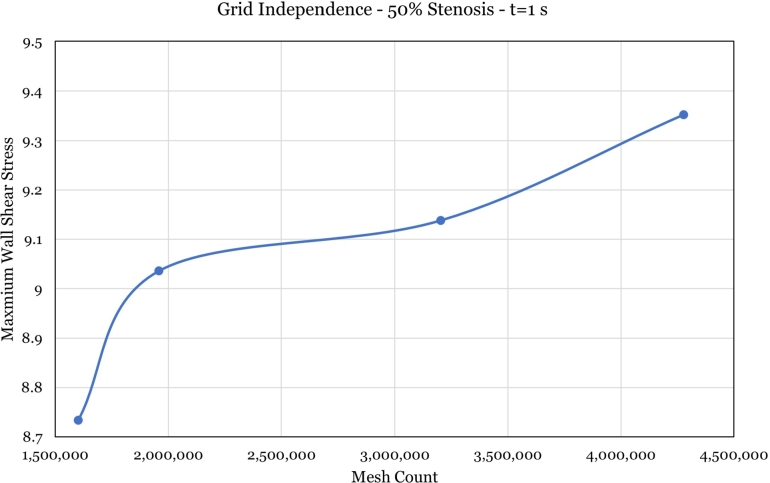
Maxmium wall shear stress for case of 50% stenosis vs Mesh count. Values extracted at t=1 s.

The interior of the aorta is meshed using the ANSYS Mechanical module. Tet4 and Hex8 elements are employed; see Fig. 7 (b) with inset (a) showing the inflation layer. As stenotic percentage changes, its diameter changes, so the modeled flow domain geometry changes slightly from one case to the other. Therefore, on average, the number of cells in the mesh is approximately 3.2 million. To arrive at this number, a mesh-independence study was conducted for the case of 50 percent stenosis at t=1 sec., varying the mesh size from 1,601,338, to 1,958,593 to 3,202,676 to 4,277,556 cells. It was found that variations in maximum wall shear stress between the latter two mesh sizes are limited to 3.7 percent (see the Appendix), confirming the convergence of the selected mesh Fig. A.1. The resulting mesh quality criteria are shown in Table 2, for the case of 50 percent stenosis.

**Table 2:** Mesh metrics for the case of 50 percent stenosis.

Metric	Mesh Average			
Aspect Ratio	2.2			
Skewness	0.19			
Orthogonal Quality	0.805			

### 2.2 CFD simulation

The simulations were conducted on workstations with 24 AMD Opteron (6174) CPUs and 64 GB of RAM. The simulations were performed on ANSYS Fluent, Release 22.0R1 [59]. A total of 10,000 time steps per cardiac cycle (1 second long) were used. The *SIMPLE* pressure-velocity coupling scheme was chosen with the bounded second-order implicit transient formulation. Iterations were carried out until the residuals in the mass continuity and momentum equations reached $10^{\text{-4}}$. A typical simulation of a full cardiac cycle lasted approximately 20 hours, or less. Five cardiac cycles were modeled for sample stenosis cases. It was confirmed that pulsatile flow behavior starting from cycle two is repetitive. Thus, the hemodynamic parameters of cycle two are herein reported for all simulations.

The following three configurations of the aorta were modeled. The first configuration features the ascending aorta and the aortic arch, while applying systolic pressure at the outlets. The second configuration features the same geometry, while applying Windkessel boundary conditions at the outlets. The third configuration includes the descending aorta into the modeled domain, also applying Windkessel boundary conditions. While it is this third configuration that is of greatest interest (the most physiologically representative), the first two configurations serve to interpret the contribution of the boundary conditions and the descending aorta to the evolving solution and its flow structures. Thus, these three configurations were solved for in a stenosis-free case, and in the six stenotic cases.

### 2.3 Post-processing (acoustic signal generation)

**Figure 8:**
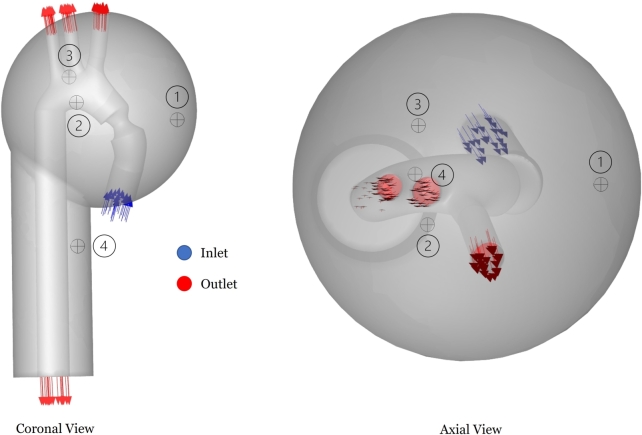
Receiver locations in the acoustic domain (modeled as static air). Receiver two is in the vicinity of the second right intercostal space. Receiver four is downstream the descending aorta.

The Ffowcs Williams-Hawkings (FW-H) method is one of four methods on ANSYS that can be used to generate an acoustic time-dependent signal [68]. FW-H allows capturing sound signals at specific locations, consistent with the notion of stethoscopic receivers. Four receivers are selected whose locations are illustrated in Fig. 8. The aim is to confirm whether the clinically preferred receiver location (right intercostal space) yields a better acoustic signal for SVAS analysis.

The domains shown in Fig. 8 can be divided into two distinct regions that correspond to blood-flow domain, and the surrounding domain where the propagation of sound off the walls of the blood vessels is computed. These walls are the source location in the FW-H equation (Equation (9)), while the acoustic field is static air in the model. Thus, the effect of the surrounding tissue and of wall elastance is ignored in this study. The FW-H equation derives from an inhomogeneous wave equation and is based on the Lighthill-analogy [68],

(9) $$\frac{1}{a_{0}^{2}}\frac{\partial^{2}p^{\prime }}{\partial t^{2}}-\nabla^{2}p^{\prime }=\frac{\partial^{2}}{\partial x_{i}\partial x_{j}}\left\{T_{ij}H(f)\right\}-\frac{\partial }{\partial x_{i}}\left\{\left[P_{ij}n_{j}+\rho u_{i}\left(u_{n}-v_{n}\right)\right]\delta (f)\right\}+\frac{\partial }{\partial t}\left\{\left[\rho_{0}v_{n}+\rho \left(u_{n}-v_{n}\right)\right]\delta (f)\right\},$$

where $u_{i}$ designates the fluid's velocity in the direction $x_{i}$, $u_{n}$ the fluid's velocity normal to the vessel's surface, $v_{i}$ the surface velocity in the direction $x_{i}$, $p^{\prime }$ is the sound pressure at the far field, $P_{ij}$ is the compressive stress tensor, and $v_{n}$ the velocity normal to the surface. Equation (9) also features the Heaviside step function H(f) and the Dirac delta function δ(f) to single out the vessel's walls (acoustic source), where *f* denotes the function describing the walls. The resultant time signal at each receiver is then obtained and transformed to the frequency domain using the Fast Fourier Transform (FFT) on MATLAB for further analysis.

## 3 Results and discussion

In this section, the contributions of the modeled outlet boundary conditions and of the descending aorta in terms of their necessity to capture the desired structures of flow are first clarified, comparing the present findings to the literature. The acoustic signals collected at the four receivers are then examined and the preferred receiver confirmed and selected to obtain a clinically relevant correlation between stenosis percentage and a newly identified acoustic frequency within the preferred auscultation range (70 Hz - 120 Hz).

### 3.1 Capturing the vortical structures in 3D

**Figure 9:**
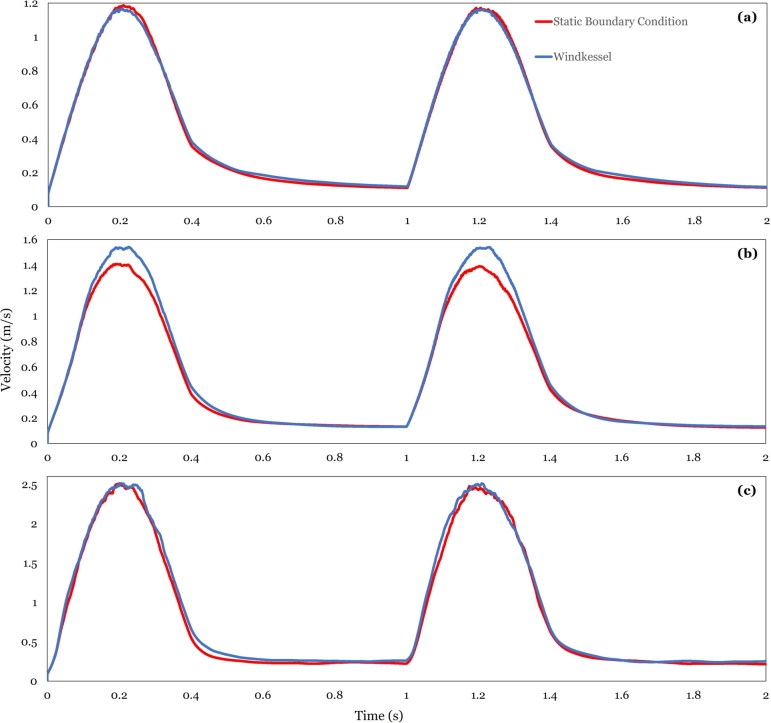
Velocity average in the ascending aorta (Fig. 5 (a)). Here, (a), (b), and (c) represent 30 percent, 50 percent and 70 percent stenosis cases respectively. It is noted that the Windkessel model only mildly improves the predictions of the average velocity relative to the static boundary conditions for case (b).

A simulation of the ascending aorta and the aortic arch is first run as shown in Fig. 5(b), i.e., excluding the descending aorta. A pulsatile velocity waveform at the inlet is used, and a static pressure at the outlet of 80 mmHg. The result is then compared to the same geometry but replacing the outlet boundary conditions with the Windkessel model. It is noted that the global hemodynamic parameters, e.g., area weighted velocity and pressure averages, remain essentially unchanged with changing boundary conditions, for the three stenotic cases tested; see Fig. 9 (a), (b) and (c) for an illustration. However, a significant reduction in computational time is achieved when the Windkessel model replaces the static pressure boundary conditions, on average 30 percent reduction. In addition, [69] noted that the Windkessel model is able to capture the flow split in the bifurcations, as opposed to the zero-pressure outlet condition, and reproduces physiological aortic pressure waveforms. As such, the Windkessel model is preferred for the remainder of this study. The Windkessel subroutine was herein developed in-house in C-language and integrated as a UDF into ANSYS Fluent.

**Figure 10:**
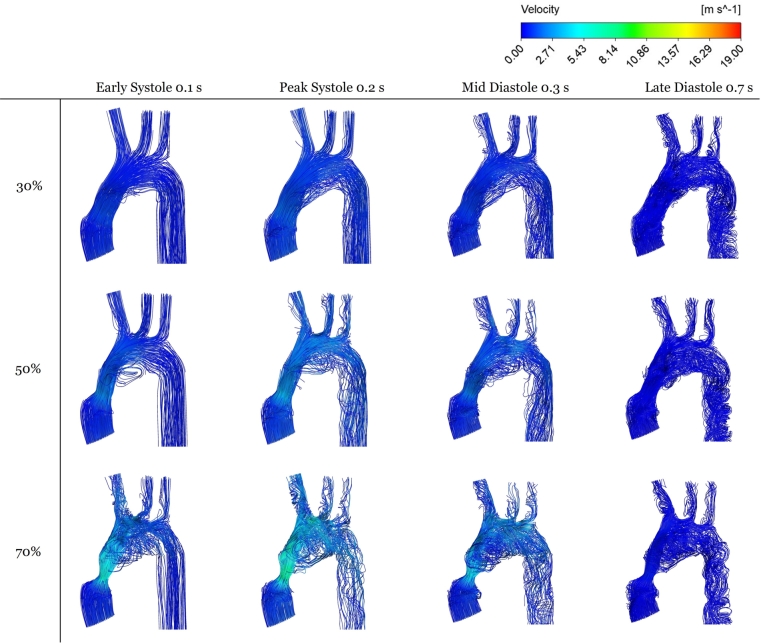
Velocity streamlines for 30, 50, and 70 percent stenosis at the corresponding time instants indicated in Fig. 6.

The resulting flow structures are shown in Fig. 10. For high stenosis, the flow is dominated by a jet emanating from the stenotic region, consistent with the literature (Zhu et al. [57], [55]). This jet-dominated flow renders a distinction between the evolving vortical structures rather difficult across the modeled domain. By adding the descending aorta to the geometry, the flow fully develops, and clearer vortical structures could be distinguished across the modeled domain in systole. The proposed strategy of adding the descending aorta to the domain is inspired by the findings of Kilner et al. [50] mentioned in the introduction.

**Figure 11:**
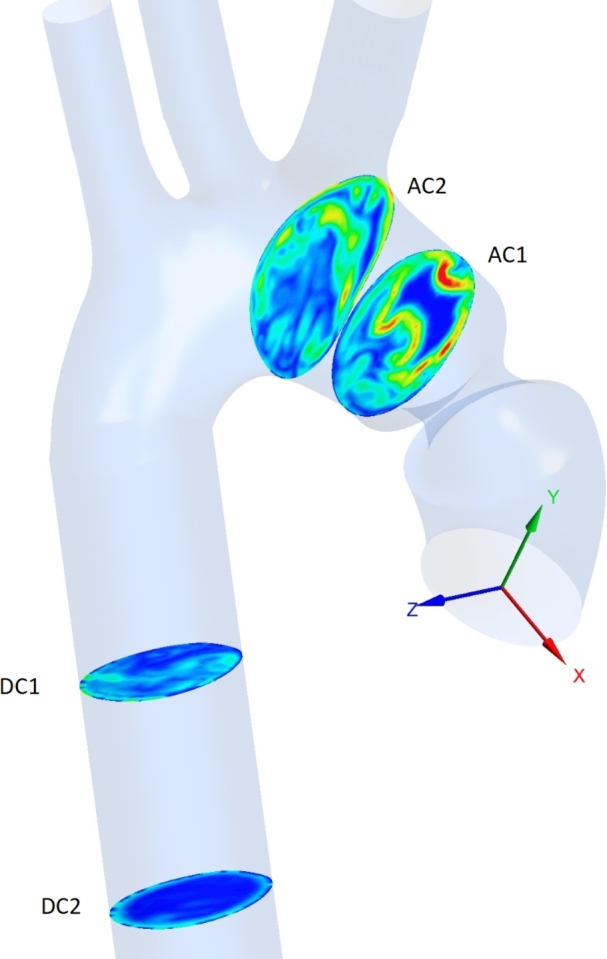
The four sections where the vorticity shown in Fig. 12 is analyzed.

**Figure 12:**
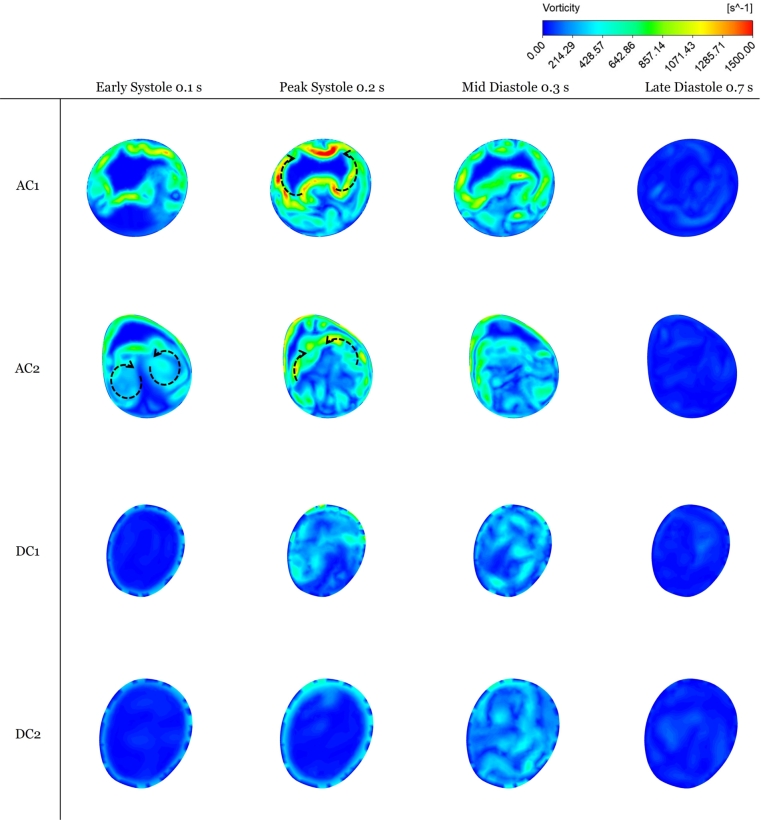
2D vorticity contours at four sections inside flow domain at chosen time instants.

Even with the descending aorta added, the velocity streamlines remain difficult to inspect visually. Hence, it was preferred to examine the vorticity contours, both in the ascending and descending aortas. Two cross-sections in the ascending aorta (AC1, AC2) were selected, and two others in the descending aorta (DC1, DC2), as shown in Fig. 11 for 50% stenosis. This procedure is akin to that performed in the literature (Zhu et al. [57], [55], Seo et al. [49]). Vortical structures are identified from the 2D vorticity contours in the transverse plane, as illustrated in Fig. 12. Specifically, during systole, *vortex shedding* occurs mainly in the ascending aorta Fig. 12. The timings, locations, and details of the vortex shedding agree with those mentioned in these reports. Also, the timing of post-stenotic vortex shedding at systole is consistent with the 2D earlier study [56]. Nevertheless, Fig. 12's confinement to the transverse plane, implies it cannot be identified with the KVS, nor its acoustic signature be matched to the degree of stenosis as per that study.

**Figure 13:**
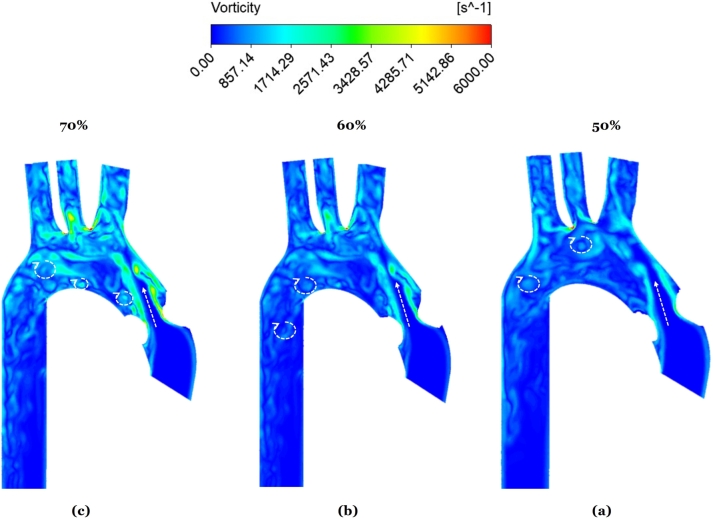
Longitudinal vorticity contours for the cases of 50%, 60% and 70% stenosis at peak systole. The jet's direction and the vortical structures are marked by arrows.

The vorticity contours in the longitudinal plane were thus further examined, as shown in Fig. 13 (a), (b) and (c) at peak systole, for the cases of 50%, 60% and 70% stenosis. The jet's domination of the flow just behind the stenosis can be easily seen in this longitudinal view, and the many vortices that form during systole in the aortic arch (to the left of the jet) are also visible. Therefore, it is to be anticipated that these stenosis-dependent changes in vortex size, distribution and intensity (in the longitudinal and transverse planes) will induce significant changes in the acoustic signature of each stenotic case, which is explored next.

### 3.2 Acoustic signal analysis

The flow-generated acoustic signal can be collected using the FW-H approach at predetermined receiver locations within the acoustic domain surrounding the stenosed artery. Three receivers are located in the vicinity of the ascending aorta and the aortic arch, while the fourth is located near the descending aorta Fig. 8. Normally, the location wherefrom clinicians listen to bruits is near the aortic valve, or where receiver two is located (the right intercostal space). The other receivers have been added in this study to see if there is a difference captured that justifies this clinical choice of location.

As noted in the previous subsection, the KVS phase cannot be singled out in any of the longitudinal or transverse sections visually inspected, since it loses its coherence in 3D [49]. Accordingly, the acoustic range of 70 - 120 Hz [61] is herein pre-selected, as recommended for stethoscopy. Moreover, note that in this study the acoustic signal for the entire thoracic aorta is extracted, not only for the post-stenotic region (as was done in the earlier 2D study). A signal transformation is then performed using FFT, applying Hanning filtering. Each acoustic signal's mean is subtracted off from the wave to reduce signal noise.

**Figure 14:**
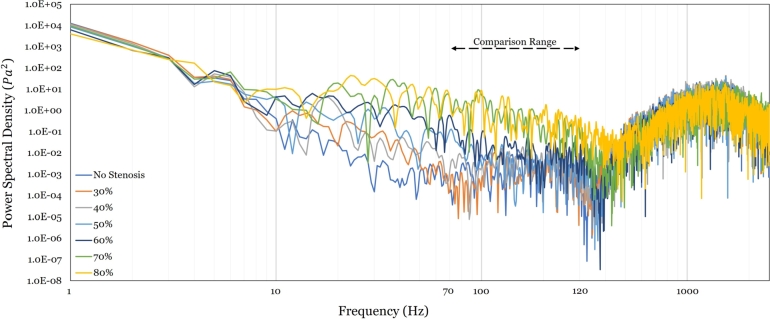
Log-Log graph for the acoustic signal obtained at receiver two for the different stenotic cases modeled. The frequency range of stethoscopic interest is highlighted between 70 and 120 Hz.

**Figure 15:**
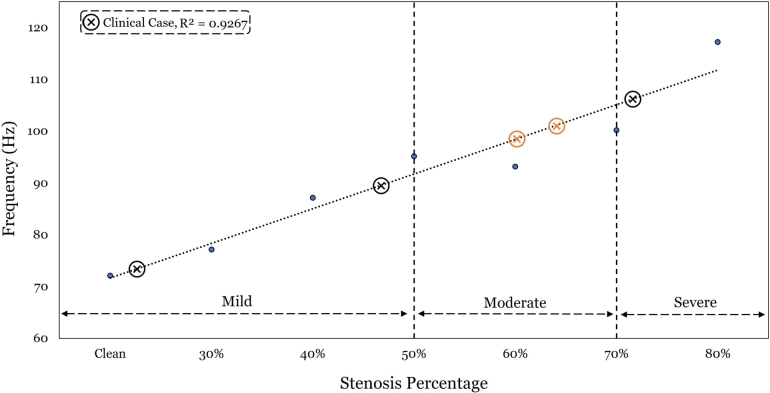
Frequency of minimum amplitude obtained from Fig. 14 for each stenosis case within the 70 - 120 Hz range. Limits of severity are based on the NHS classification [73].

Acoustic results from receivers one, three and four were found indeed to exhibit poor sensitivity to degree of stenosis; i.e., any correlation that could be constructed yielded a rather low correlation coefficient. Not so for receiver two, which aligns with the clinical preference for it. Fig. 14 shows the frequency spectrum at receiver two per degree of stenosis. The frequency ranges from 1 Hz (assumed heart rate) to beyond 1,000 Hz. On the Figure, the range of stethoscopic interest is also demarcated (70 - 120 Hz). It is further seen that the acoustic signals at different stenosis degrees most visibly diverge from one another within the frequency range of 10 Hz to 120 Hz, which contains the recommended stethoscopic range. This finding is expected to yield a strong correlation between the acoustic frequency from the stethoscopic range and the degree of stenosis (in comparison to the break frequency in the literature, which is only weakly sensitive to stenotic variation degrees). Indeed, it is here found that the frequency of *minimum amplitude* within this stethoscopic range generates a linear scaling law, see Fig. 15.

**Figure 16:**
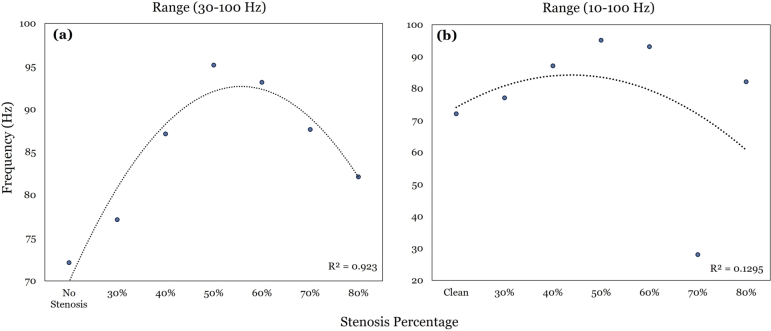
(a) represents correlation between frequency of minimum amplitude in the 30-100 Hz range vs. stenosis percentage, (b) is similar to (a) but for the 10-100 Hz range.

To confirm computationally the optimality of the 70 - 120 Hz stethoscopic range reported clinically, candidate correlations from beyond this range are here explored, to check whether their resulting scaling laws are indeed worse than the linear one offered by this preferred range. Indeed, various possible ranges of frequencies have been suggested as relevant in the literature, e.g., (Charbonneau et al. [70], Rennert et al. [71], Spencer et al. [72]). Thus, the ranges 10 - 100 Hz, 30 - 100 Hz, in addition to the 70 - 120 Hz are searched for scaling laws. The former two ranges yield less-strong correlations (of lower rank coefficients), that are essentially non-linear, with non-unique representations of a degree of severity, see Fig. 16 (a), (b). Thus, the optimal range for auscultating bruits is confirmed to indeed lie in the 70 - 120 Hz range.

For further validation, five clinical stethoscopic signals from online databases (e.g., [74], and [75]) are acquired. The five clinical cases have been assessed by an experienced cardiologist (using a stethoscope) as severe, moderate or mild. The exact percentages are unknown, since cardiologist cannot guess them. Nevertheless, the boundaries for severe, moderate and mild stenosis are quantitatively marked (by percent stenosis) by the NHS [73]. Hence, the five clinical signals are transformed using FFT to see where they lie relative to those NHS-classified boundaries. Using the scaling law obtained in Fig. 15, the assessment of the cardiologists relative to the NHS classification for three of the cases is confirmed; two mild, and one severe (i.e., the extreme cases are correctly assessed by clinicians). For the two remaining clinical cases, highlighted by orange markers on the Figure, the cardiologist's assessment was severe. However their auscultated frequencies fall roughly 5% short of the severe boundary, which the linear scaling law more accurately categorizes as moderate cases. The proposed scaling law thus can serve as a quantitative tool in support of cardiologists to better determine the degree of stenosis, especially for non-extreme cases.

## 4 Conclusion

In this study, a linear scaling law that correlates stethoscopically auscultated acoustic frequencies (70 - 120 Hz) to the degree of stenosis in 3D is identified, using anatomically representative geometries and LES-FFT computational modeling. The benefit of applying Windkessel outlet boundary conditions (physiologically representative) is primarily to accelerate LES computational convergence. The study further confirms, by comparing scaling laws, the preferability of the receiver located at the right intercostal space to listen for bruits, consistent with clinical practice for SVAS. Five clinical stethoscopic signals were used to verify the scaling law in relation to the NHS classification of stenosis degrees, and the assessments of experienced cardiologists. Three of those clinical assessments are confirmed directly. It is further shown that for two clinical cases the cardiologists conservatively assessed them as severe, though their auscultated frequencies were roughly 5% away from the corresponding NHS boundary (per the newly identified scaling law), and should thus be classified as moderate cases of stenosis. Such a range of error in (qualitative) professional judgment is to be expected (Dhuper et al. [24], Mangione et al. [25]), which motivated this study in the first place. As such, the scaling law can serve as a quantitative decision-support tool for clinicians to more reliably interpret stethoscopic auscultations and accurately assess the degree of SVAS in their patients. Investigations of other stenotic geometries (beyond the SVAS hourglass studied herein) would enhance the clinical relevance of the proposed approach, and would be pursued in future research.

## CRediT authorship contribution statement

**Ahmed M. Ali:** Data curation, Formal analysis, Investigation, Validation, Visualization, Writing – original draft. **Aly A. Ghobashy:** Data curation, Formal analysis, Investigation, Validation, Visualization, Writing – original draft. **Abdelrahman A. Sultan:** Data curation, Formal analysis, Investigation, Validation, Visualization, Writing – original draft. **Khalil I. Elkhodary:** Conceptualization, Formal analysis, Funding acquisition, Investigation, Project administration, Resources, Software, Supervision, Writing – original draft, Writing – review & editing. **Mohamed El-Morsi:** Conceptualization, Formal analysis, Funding acquisition, Methodology, Project administration, Supervision, Writing – review & editing.

## Declaration of Competing Interest

The authors declare that they have no known competing financial interests or personal relationships that could have appeared to influence the work reported in this paper.

## Data Availability

Data will be made available on request.
